# Lower synovial leucocyte count and polymorphonuclear percentage reliably differentiate periprosthetic joint infection after unicompartmental knee arthroplasty

**DOI:** 10.1002/ksa.70036

**Published:** 2025-09-04

**Authors:** Stefanie Donner, Georg Matziolis, Yves Gramlich, Igor Lazic, Daniel Schrednitzki, Anne Pohrt, Nora Renz, Nils Meißner

**Affiliations:** ^1^ Center for Musculoskeletal Surgery Charité – Universitätsmedizin Berlin Berlin Germany; ^2^ Orthopedic Department Waldkliniken Eisenberg University Hospital Jena, FSU Jena, Campus Eisenberg Eisenberg Germany; ^3^ Agaplesion Markus Krankenhaus Frankfurt Germany; ^4^ BG Unfallklinik Frankfurt Frankfurt Germany; ^5^ Clinic of Orthopedics and Sports Orthopedics Klinikum Rechts der Isar Technische Universität München München Germany; ^6^ Department of Orthopaedic and Trauma Surgery Sana Hospital Lichtenberg Berlin Germany; ^7^ Insitute of Biometry and Clinical Epidemiology Charité – Universitätsmedizin Berlin Berlin Germany; ^8^ Department of Infectious Diseases, Inselspital Bern, Bern University Hospital University of Bern Switzerland; ^9^ Department of Orthopaedic Surgery Sana Hospital Sommerfeld Kremmen Germany

**Keywords:** diagnostic criteria, periprosthetic joint infection, synovial fluid leucocyte count, synovial polymorphonuclear leucocytes percentage, unicompartmental knee arthroplasty

## Abstract

**Purpose:**

This study aimed to determine diagnostic thresholds for synovial fluid leucocyte count and polymorphonuclear (PMN) percentage to identify the diagnosis periprosthetic joint infection (PJI) in patients with failed unicompartmental knee arthroplasties (UKAs).

**Methods:**

This multicentre retrospective cohort study included 239 patients who underwent revision of an UKA for either septic or aseptic indications at five university‐affiliated medical centres. Among these, 30 patients (13%) underwent revision for PJI and 209 (87%) for noninfectious causes. PJI was diagnosed according to the European Bone and Joint Infection Society (EBJIS) criteria. Preoperative synovial fluid leucocyte count, synovial PMN percentage, serum C‐reactive protein (CRP) and white blood cell (WBC) count were evaluated. Diagnostic performance and optimal thresholds for each parameter were assessed using receiver operating characteristic curves and Youden's index.

**Results:**

The PJI group demonstrated significantly higher median synovial leucocyte counts (11,399/μL vs. 429/μL, *p* < 0.001), and significantly higher synovial PMN percentage (82% vs. 28%, *p* < 0.001) compared to the non‐PJI group. The optimal diagnostic cut‐off for synovial fluid leucocyte count was 2318/μL (area under curve [AUC] 0.93; sensitivity 83%, specificity 95%) and for PMN percentage, 64% (AUC 0.90; sensitivity 76%, specificity 95%). Serum CRP (cut‐off 9 mg/L; AUC 0.85) and WBC count (cut‐off 8 G/L; AUC 0.71), showed lower diagnostic accuracy.

**Conclusions:**

This study establishes UKA‐specific diagnostic thresholds for PJI, which are consistent with the EBJIS PJI criteria established for TKA. Synovial biomarkers, particularly synovial fluid leucocyte count and PMN percentage, demonstrated superior diagnostic performance compared to serum CRP and WBC count. These findings underscore the need for tailored diagnostic criteria to improve the accuracy of PJI diagnosis and guide clinical decision‐making in UKA revision.

**Level of Evidence:**

Level III.

**Abbreviations**
AUC: area under curve
CI: confidence interval
CRP: C‐reactive protein
EBJIS: European Bone and Joint Infection Society
ESCMID: European Society of Clinical Microbiology and Infectious Diseases
ICM: international consensus meeting
MSIS: Musculuskelatal Infection Society
OPC: operation procedure code
PJI: periprosthetic joint infection
PMN: polymorphonuclear
ROC: receiver operating characteristics
TJA: total joint arthroplasty
TKA: total knee arthroplasty
UKA: unicompartmental knee arthroplasty
WBC: white blood cell

## INTRODUCTION

Unicompartmental knee arthroplasty (UKA) is an established and cost‐effective treatment option for isolated medial or lateral compartment osteoarthritis, offering favourable clinical outcomes and high patient satisfaction [6, 8, 19]. Although rare, periprostethic joint infection (PJI) following UKA remains a serious complication associated with significant morbidity and mortality [4, 14, 15]. While established definitions and criteria for PJI exist in total joint arthroplasty (TJA) by the Infectious Diseases Society of America (IDSA), the Musculoskeletal Infection Society (MSIS) and the European Bone and Joint Infection Society (EBJIS), UKA specific criteria remain undefined [7, 10, 11, 12, 13].

Diagnosis of PJI relies on a combination of clinical, radiological and laboratory findings, with synovial fluid analysis serving as a key diagnostic tool. Although synovial leucocyte count and polymorphonuclear (PMN) percentage are validated in total knee arthroplasty (TKA), limited evidence exists for their applicability in UKA. Given the reduced implant burden and reduced synovial surface of UKAs, it is hypothesised that the associated inflammatory response in PJI may be less pronounced, potentially resulting in lower synovial fluid leucocyte counts and PMN percentages.

Only two previous studies have investigated UKA‐specific serologic and synovial fluid thresholds, both limited by small sample sizes and incomplete data [1, 18]. Therefore, this study aimed to define diagnostic thresholds for synovial fluid and routine preoperative laboratory markers in patients undergoing revision UKA for suspected PJI, with an emphasis on synovial leucocyte count and PMN percentage.

## MATERIALS AND METHODS

This retrospective multicenter cohort study included patients who underwent either septic or aseptic revision of medial or lateral UKA at five university‐affiliated hospitals specialised in knee arthroplasty. Patients were identified by their operation procedure codes (OPCs) and by reviewing institutional electronic medical records. Each case was evaluated by an interdisciplinary team comprising an orthopaedic surgeon and an infectious diseases specialist, and subsequently categorised into either a PJI group (*n* = 30) or a non‐PJI group (*n* = 209). Medial (236 UKAs; 99%) and lateral (3 UKAs; 1%) UKAs were analysed together. The final cohort consisted of 140 women (59%) and 99 men (41%), with a mean age of 67 years (range, 45–92 years). The mean time from the primary UKA implantation to revision was 5 years (range, 0–21 years). Ethical approval was obtained from the institutional ethics committee of all participating study centres. Demographic data are shown in Table 1.

**Table 1:** Demographic and clinical characteristics of included patients.

Variable		Non‐PJI (*n* = 209)	PJI (*n* = 30)	*p* value
Sex^a^	Female	125 (60)	16 (50)	0.550
	Male	84 (40)	14 (50)	
Patient age^b^ (years)		67 (45−92)	65 (48−83)	0.151
Duration of symptoms^a^	<1 month	5 (2)	5 (17)	0.001
	>1 month	205 (98)	25 (83)	
Prosthetic loosening^a^		69 (33)	9 (30)	0.117
Sinus tract^a^		0 (0)	1 (3)	0.245
Fever^a^		0 (0)	1 (3)	0.245

Abbreviation: PJI, periprosthetic joint infection.

^a^ The values are given as the number of cases, with the percentage of cases in parentheses.

^b^ The values are given as the means, with the range in parentheses.

### Study population

All patients aged >18 years who underwent revision from UKA to TKA were included. Patients were categorised into a PJI group and a non‐PJI group. Non‐PJI indications for revision UKA included instability, progression of osteoarthritis in other compartments, or aseptic loosening leading to either one‐stage oder two‐stage revision procedures. Patients with incomplete datasets, a history of prior PJI in the same knee, or revision of patellofemoral arthroplasties were excluded. PJI was suspected based on clinical signs of inflammation in the knee, including joint pain, erythema, soft tissue swelling, joint effusion, wound discharge or restricted range of motion. In the PJI group, microorganisms were identified from synovial fluid, intraoperative tissue samples and in sonication fluid, where available, and subsequently classified as positive or negative according to previously published criteria [16].

### PJI definition

PJI was confirmed according to the EBJIS criteria [7]. Infections were classified as acute if they occurred within 4 weeks of the most recent surgery or presented with a symptom duration of less than 4 weeks. All remaining cases were categorised as chronic. Cases initially classified as ‘infection likely’ were independently reviewed by the authors and reclassified as either PJI or non‐PJI based on consensus. Synovial fluid parameters, including leucocyte count and the PMN percentage were excluded as confirmatory diagnostic criteria, as they represented primary study variables.

### Statistical analysis

Optimal cut‐off values were determined using the Youden index. Data from patients who underwent revision for PJI after UKA and non‐PJI revisions were analysed separately. Comparisons between groups were performed using appropriate statistical tests depending on scale level and distribution. For normally distributed continuous data, the Student's *t*‐test was applied. For nonnormally distributed data, the Wilcoxon–Mann–Whitney test was used. Categorial variables were compared using the chi‐squared test.

Receiver operating characteristic (ROC) curves were constructed to evaluate synovial fluid leucocyte count, synovial PMN percentage, serum CRP and white blood cell (WBC) count differential in diagnosing PJI. The area under the curve (AUC) was computed to quantify diagnostic accuracy. Confidence intervals (CIs) were computed using the method described by Obuchowski et al. [9], accounting for the clustered design of this multicenter study.

**Figure 1:**
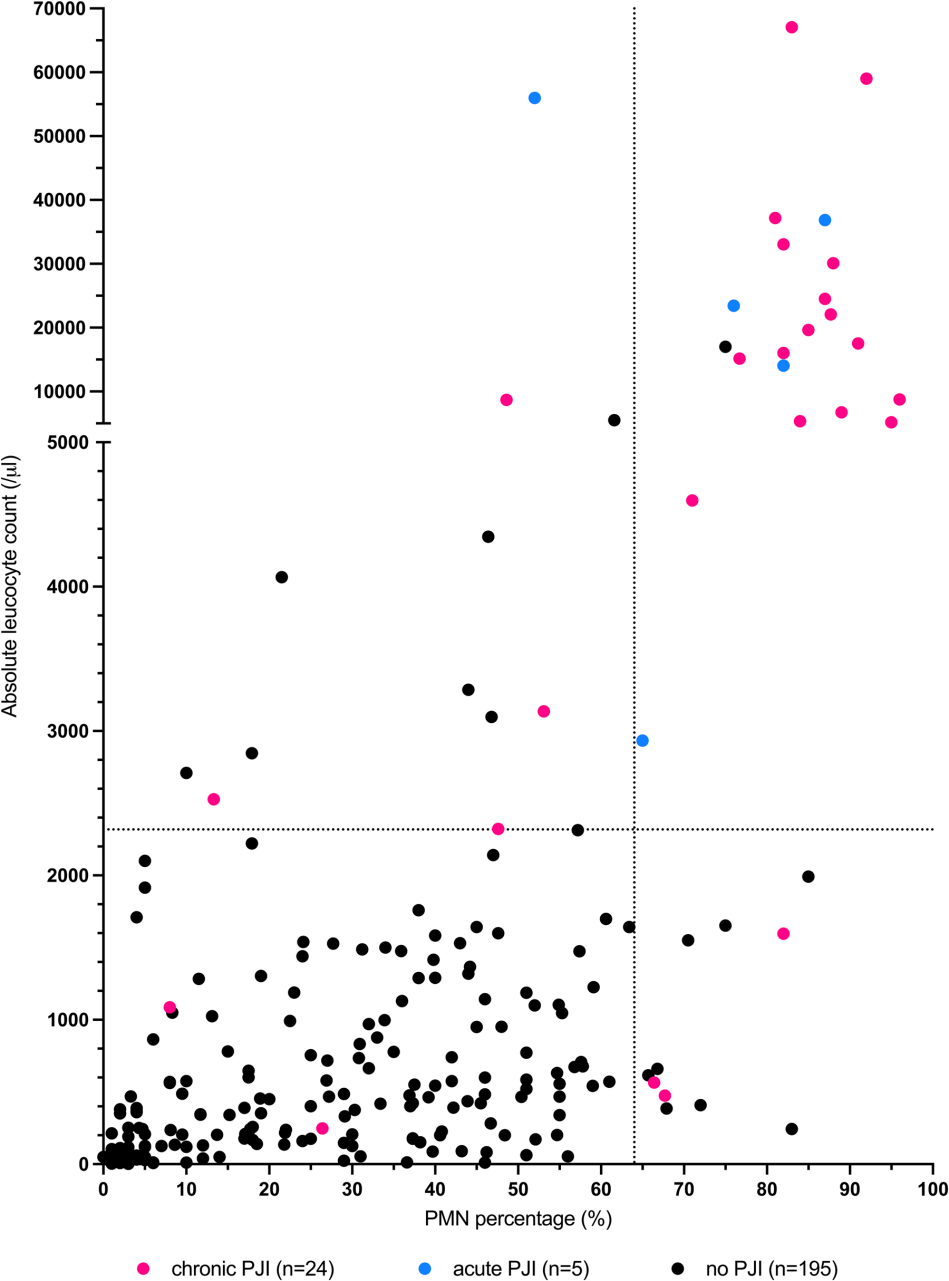
Analysis of patients with both absolute leucocyte count and PMN percentage determined in synovial fluid. Dotted lines indicate the proposed cut‐offs based on our results. PJI, periprosthetic joint infection; PMN, polymorphonuclear.

Sensitivity, specificity, positive and negative predictive values were calculated for each cut‐off. A *p*‐value < 0.05 was considered statistically significant. All analyses were performed using the R version 4.3.0 (R Foundation for Statistical Computing). Figure 1 was generated using Prism (version 10.3.0; GraphPad).

## RESULTS

### Microbiological findings and diagnostic markers

In the PJI group, cultures were positive in 17 patients (57%), with *Staphylococcus* species being the most commonly identified pathogens (Table 2). The median values for synovial fluid leucocyte counts and PMN percentages for patients were significantly elevated in patients with PJI compared those in the non‐PJI group (Table 3). The median synovial fluid leucocyte count was 11,399 cells/μL (range, 248–67,060 cells/μL) in the PJI group and 429 cells/μL (range, 2–17,000 cells/μL) in the non‐PJI group (*p* < 0.001). The median PMN percentage was 82% (range; 8%–96%) compared to 28% (range, 1%–85%) for the PJI‐group and non‐PJI group, respectively (*p* < 0.001; Figure 1).

**Table 2:** Causative microorganisms of periprosthetic joint infection.

Pathogen	Culture results (%)
Monomicrobial infections	15 (50)
Coagulase‐negative staphylococci^a^	7
*Staphylococcus aureus*	2
*Streptococcus* species^b^	2
*Cutibacterium acnes*	1
*Escherichia coli*	1
*Enterococcus faecalis*	1
*Roseomonas mucosa*	1
Polymicrobial infections	2 (7)
Coagulase‐negative staphylococci^c^	2
*Streptococcus dysgalactiae*	1
*Escherichia coli*	1
Culture‐negative infections	13 (43)

*Note*: The values are given as the number of cases, with the percentage of cases in parentheses. Abbreviation: PJI, periprosthetic joint infection.

^a^ Including *S. epidermidis* (4), *S. lugdunensis* (*n* = 1), *S. warneri* (*n* = 1), *S. schleiferi* (*n* = 1).

^b^ Including *S. dysgalactiae* (*n* = 1), *S. parasanguinis* (*n* = 1).

^c^ Including *S. epidermidis* (*n* = 1), no further identification (*n* = 1).

**Table 3:** Comparison of different diagnostic tests between non‐PJI and PJI group.

Diagnostic test	Non‐PJI (*n* = 209)^a^	PJI (*n* = 30)^a^	*p* value
Synovial fluid leucocyte count (cells/μL)	429 (2−17,000)	11,399 (248−67,060)	<0.001
PMN percentage (%)	28 (1–85)	82 (8–96)	<0.001
CRP (mg/L)	2.0 (0.3–22.0)	16.2 (0.3−292.4)	<0.001
WBC count (G/L)	6.6 (2.5–12.1)	8.6 (4.7−14.6)	<0.001

Abbreviations: CRP, C‐reactive protein; PJI, periprosthetic joint infection; PMN, polymorphonuclear; WBC, white blood cell.

^a^ The values are given as the median, with the range in parentheses.

The median serum CRP was 16.2 mg/L (range, 0.3–292.4 mg/L) in the PJI group compared to 2.0 mg/L (range, 0.3–22.0 mg/L) in the non‐PJI group (*p* < 0.001). The median WBC count was 8.6 G/L (range, 4.7–14.6 G/L) compared to 6.6 G/L (range, 2.5–12.1 G/L) for the PJI group and non‐PJI group, respectively (*p* < 0.01; Table 3).

### Diagnostic performance of markers

**Figure 2:**
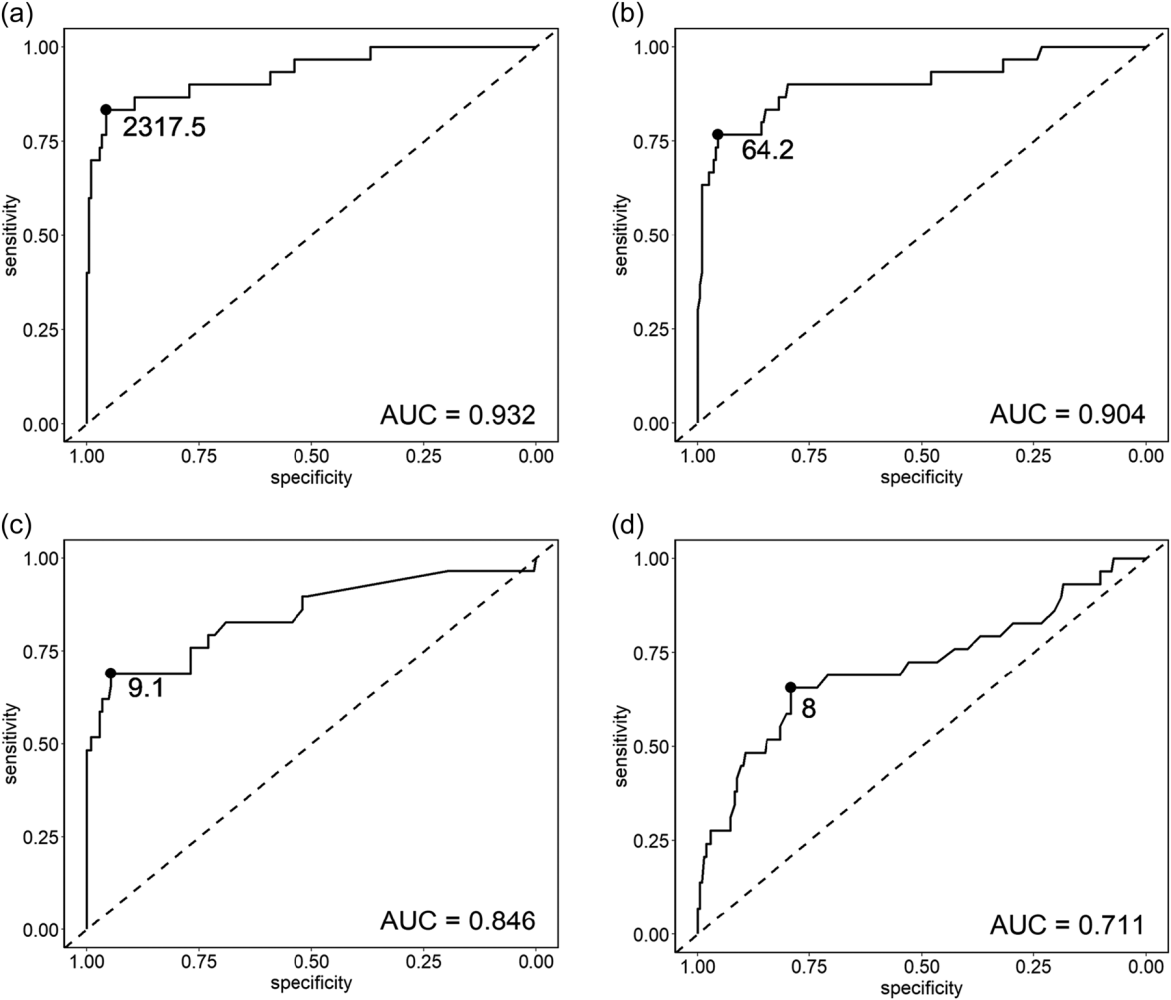
Receiver operating characteristics (ROC) curves for (a) synovial fluid leucocyte count, (b) PMN percentage, (c) serum CRP, (d) WBC count. Supporting table: Distribution of PJI and non‐PJI cases per study center. AUC, area under the curve; CRP, C‐reactive protein; PMN, polymorphonuclear; WBC, white blood cell.

The AUC for synovial fluid leucocyte count was 0.93, and the AUC for synovial PMN percentage was 0.90 (Figure 2; Table 4). In comparison, the AUCs for serum CRP and WBC count were lower, at 0.85 and 0.71, respectively (Figure 2; Table 4). The optimal cut‐off value for synovial fluid leucocyte count was 2318 cells/μL (83% sensitivity and 96% specificity), and 64% for synovial PMN percentage (77% sensitivity and 96% specificity; Figure 2; Table 4). Additionally, the computed cut‐offs for serum CRP were 9.1 mg/L (69% sensitivity and 95% specificity) and for WBC count 8.0 G/L (66% sensitivity and 79% specificity; Figure 2; Table 4).

**Table 4:** Diagnostic performance of diagnostic markers.

Diagnostic test	Cut‐off value	Sensitivity^a^	Specificity^a^	AUC ROC^a^	PPV	NPV
Synovial fluid leucocyte count	2318 cells/μL	0.833 [0.70, 0.967]	0.956 [0.927, 0.981]	0.932 [0.830, 1.033]	0.742	0.976
	>1500 cells/μL	0.867 [0.733, 0.967]	0.859 [0.806, 0.908]		0.469	0.978
	>3000 cells/μL	0.767 [0.6, 0.9]	0.966 [0.942, 0.99]		0.764	0.966
Synovial fluid PMN percentage	64.2%	0.767 [0.60, 0.90]	0.955 [0.924, 0.98]	0.904 [0.785, 1.024]	0.708	0.966
Serum C‐reactive protein	9.05 mg/L	0.69 [0.482, 0.828]	0.946 [0.912, 0.975]	0.864 [0.757, 0.934	0.647	0.955
White blood cell count	8.02 G/L	0.655 [0.482, 0.828]	0.791 [0.733, 0.84]	0.711 [0.648, 0.775]	0.311	0.941

Abbreviations: AUC, area under the curve; PMN, polymorphonuclear; ROC, receiver operating characteristic.

^a^ The values are presented with the upper and lower limits of the 95% confidence intervals in parentheses.

## DISCUSSION

The most important finding of the present study was that synovial fluid leucocyte count demonstrated the highest diagnostic accuracy for diagnosing PJI following UKA, with excellent sensitivity and a proposed threshold of 2318 cells/μL. PJI remains a devastating complication following UKAs, necessitating precise and early diagnosis for effective treatment. While diagnostic thresholds for standard tests have been well established for PJI in TKAs, corresponding data for UKA are scarce, making it crucial to establish UKA‐specific cut‐offs. This cohort represents the largest dataset with complete data on this subject.

Synovial leucocyte count showed the highest AUC, sensitivity and specificity for diagnosing PJI in UKAs, followed by synovial PMN percentage, consistent with previous studies [1, 18]. In contrast, we found that due to their lower accuracy, serum CRP and WBC count play a subordinate role in the diagnosis of PJI in UKA.

Synovial fluid leucocyte cell count is one of the primary diagnostic tools for detecting PJI in TJA [2, 5, 7, 17]. In this present study, it was confirmed as the most reliable parameter for diagnosing PJI following UKA. The optimal threshold was 2318 cells/μL, with an AUC of 0.93, sensitivity of 83% and specificity of 95%. In comparison, Schwartz et al. proposed a higher threshold of 6200 cells/μL, achieving an AUC of 0.99, sensitivity of 90% and specificity of 97%. Similarly, Cohen‐Levy et al. reported a cut‐off of 2695 cells/μL, slightly higher than the threshold found in the present study, with an AUC of 0.97, sensitivity of 94% and specificity of 90% [1, 18]. However, the threshold proposed by both Schwartz et al. [18]and Cohen‐Levy et al. were based on incomplete datasets, introducing potential selection bias [1, 18].

The second most accurate cut‐off established in our study was synovial PMN percentage, with a cut‐off of 64%, achieving an AUC of 0.90, sensitivity of 76%, and specificity of 95%. Schwartz et al. reported a cutoff of 60%, achieving an AUC of 0.97, sensitivity of 91% and specificity of 94% [18]. Cohen‐Levy et al. proposed a lower cutoff of 53%, with an AUC of 0.97, sensitivity of 94% and specificity of 93% [1]. While the thresholds vary slightly, it is worth noting that Schwartz et al.'s analysis were based on only 35% of their cohort [18], and Cohen‐Levy et al.'s on less than 50% [1]. Despite these differences, all three studies, including the present study, consistently support the diagnostic value of synovial PMN percentage in UKA PJI.

Variation in established diagnostic thresholds may hypothetically be attributed to differences in symptom acuity and the virulence of pathogens identified across studies. In the present cohort, the proportion of PJIs caused by highly virulent organisms was low, as was the percentage of acute infections. This may partially explain the lower thresholds observed in this study. Consistent with this, Kheir et al. demonstrated a correlation between pathogen virulence and the degree of inflammatory response in both serum and synovial fluid markers [3]. Additionally, the high proportion of culture‐negative PJIs in this cohort may have contributed to the lower cut‐offs. It remains unclear to what extent these cases represent misclassified aseptic failures, which could further impact threshold determination.

While serum CRP and WBC count are less accurate than synovial markers, they remain useful adjuncts in diagnosing PJI, particularly when synovial fluid analysis is unavailable. In both comparative studies, CRP demonstrated lower specificity, with similar thresholds of 14 and 11 mg/L, respectively [1, 18]. Neither study proposed WBC count cut‐offs, limiting direct comparisons. Based on the findings in this study, it can not be recommended relying on WBC count as a standalone diagnostic marker for PJI in UKA.

Despite its strengths, this study has several limitations. First, the retrospective design introduces potential selection and information biases. Second, variability in diagnostic protocols across institutions may affect generalisability. Third, while this cohort represents the largest cohort with complete synovial and serum marker data in the context of UKA revision to date, the sample size of the PJI group remains relatively small due to the rarity of this complication. Lastly, differences in microbial aetiology may influence the inflammatory response, which complicates the establishment of universally applicable diagnostic thresholds.

## CONCLUSION

This study establishes UKA‐specific cut‐offs for synovial fluid leucocyte count, PMN percentage, CRP and WBC count, with synovial markers demonstrating excellent diagnostic performance. Implementing these thresholds in daily practice may help reduce diagnostic uncertainty, facilitate timely and appropriate treatment decisions and ultimately improve patient outcomes. Future research should aim to validate these thresholds in prospective cohorts and assess their utility across varied clinical settings to support their integration into standardised guidelines for PJI diagnosis in UKA.

## AUTHOR CONTRIBUTIONS

**Stefanie Donner**: Conceptualisation; methodology; formal analysis; investigation; data curation; writing—original draft preparation. **Georg Matziolis**: Data curation; writing—reviewing and editing. **Yves Gramlich**: Data curation; writing—reviewing and editing. **Igor Lazic**: Data curation; writing—reviewing and editing. **Daniel Schrednitzki**: Writing—reviewing and editing. **Anne Pohrt**: Statistical analysis; writing—reviewing and editing. **Nora Renz**: Conceptualisation; supervision; writing—reviewing and editing. **Nils Meißner**: Data curation; writing—reviewing and editing, supervision.

## CONFLICT OF INTEREST STATEMENT

The authors declare no conflicts of interest.

## ETHICS STATEMENT

The retrospective study design was approved by the local ethics board (EA4/066/24) at Charité Universitätsmedizin Berlin.

## Supporting information

Supporting table1.

## Data Availability

The data that support the findings of this study are available from the corresponding author upon reasonable request.
