# Supplementary material for: Lower synovial leucocyte count and polymorphonuclear percentage reliably differentiate periprosthetic joint infection after unicompartmental knee arthroplasty
Source: Knee Surg Sports Traumatol Arthrosc. 2025 Sep 4;34(4):1310–7. doi: 10.1002/ksa.70036 (PMC13037359; doi:10.1002/ksa.70036)
Supplement: Supplementary file 1 — Supporting table1. [file KSA-34-1310-s001.docx]

**Supplementary table1: Distribution of PJI and non-PJI Cases per study center**

| **Study center** | **Non-PJI (n=209, %)** | **PJI (n=30, %)** | **Total (n=239, %)** |
| --- | --- | --- | --- |
| 1 | 22 (10%) | 4 (13%) | 26 (11%) |
| 2 | 92 (44%) | 9 (30%) | 101 (42%) |
| 3 | 38 (18%) | 9 (30%) | 47 (20%) |
| 4 | 10 (5%) | 4 (13%) | 14 (6%) |
| 5 | 47 (22%) | 4 (13%) | 51 (21%) |

The values are presented as number of cases and percentage in parenthesis.

PJI=periprosthetic joint infection
